# Kidney Dosimetry in ^177^Lu and ^90^Y Peptide Receptor Radionuclide Therapy: Influence of Image Timing, Time-Activity Integration Method, and Risk Factors

**DOI:** 10.1155/2013/935351

**Published:** 2013-06-20

**Authors:** F. Guerriero, M. E. Ferrari, F. Botta, F. Fioroni, E. Grassi, A. Versari, A. Sarnelli, M. Pacilio, E. Amato, L. Strigari, L. Bodei, G. Paganelli, M. Iori, G. Pedroli, M. Cremonesi

**Affiliations:** ^1^Medical Physics Department, European Institute of Oncology, 20141 Milan, Italy; ^2^Medical Physics Department, IRCCS Arcispedale Santa Maria Nuova, 42123 Reggio Emilia, Italy; ^3^Nuclear Medicine Department, IRCCS Arcispedale Santa Maria Nuova, 42123 Reggio Emilia, Italy; ^4^Medical Physics Department, IRCCS Istituto Scientifico Romagnolo per lo Studio e la Cura dei Tumori, 47014 Meldola, Italy; ^5^Medical Physics Department, Azienda Ospedaliera S. Camillo Forlanini, 00151 Rome, Italy; ^6^Section of Radiological Sciences, Department of Biomedical Sciences and Morphologic and Functional Imaging, University of Messina, 98125 Messina, Italy; ^7^Laboratory of Medical Physics and Expert Systems, National Cancer Institute Regina Elena, 00144 Rome, Italy; ^8^Nuclear Medicine Department, European Institute of Oncology, 20141 Milan, Italy

## Abstract

Kidney dosimetry in ^177^Lu and ^90^Y PRRT requires 3 to 6 whole-body/SPECT scans to extrapolate the peptide kinetics, and it is considered time and resource consuming. We investigated the most adequate timing for imaging and time-activity interpolating curve, as well as the performance of a simplified dosimetry, by means of just 1-2 scans. Finally the influence of risk factors and of the peptide (DOTATOC versus DOTATATE) is considered. 28 patients treated at first cycle with ^177^Lu DOTATATE and 30 with ^177^Lu DOTATOC underwent SPECT scans at 2 and 6 hours, 1, 2, and 3 days after the radiopharmaceutical injection. Dose was calculated with our simplified method, as well as the ones most used in the clinic, that is, trapezoids, monoexponential, and biexponential functions. The same was done skipping the 6 h and the 3 d points. We found that data should be collected until 100 h for ^177^Lu therapy and 70 h for ^90^Y therapy, otherwise the dose calculation is strongly influenced by the curve interpolating the data and should be carefully chosen. Risk factors (hypertension, diabetes) cause a rather statistically significant 20% increase in dose (*t*-test, *P* < 0.10), with DOTATATE affecting an increase of 25% compared to DOTATOC (*t*-test, *P* < 0.05).

## 1. Introduction

PRRT is an important option for the treatment of neuroendocrine tumors (NETs) and other somatostatin receptor expressing neoplasms. Overall, the response rate of complete, partial, and minor response reaches 50% for Lu DOTATATE [1]. The cumulative absorbed dose to the tumor is limited by the irradiation of the organs at risk, the kidney, and the red marrow. In particular, specific and nonspecific radionuclide accumulation in the kidneys is of major concern, and considerable variation has been found in patients' maximal kidney uptake and biological washout.

In general, the biological processes are assumed to follow a first-order kinetics [2], which can be described by the sum of exponential functions. 

Renal peptide clearance is characterized by a single- or two-step phase, the first lasting about 24 hours after injection. In order to best extrapolate the renal time-activity curve, calculate the time-integrated activity $\tilde{a}$, and have a reliable estimate of the absorbed dose, several experimental data need to be collected, requiring several planar and/or 3D scans. 

When ^177^Lu is used for therapy, its gamma decay branch allows imaging at the same time: 3 to 5 whole-body/SPECT scans are taken from 2 to 7 days after the infusion. When ^90^Y is the radiopharmaceutical, either ^111^In- or ^86^Y-labeled peptides are used as a surrogate and 2 to 5 scans are collected up to 2-3 days p.i. [3]. These activity data are usually fitted by means of monoexponential functions [4–6], biexponential [3], or trapezoids [7]. 

The great value of dosimetry is an established tenet, nonetheless each experimental point requires time-consuming acquisitions. In this respect, an optimal choice of the number of scans and of their temporal location is warranted to balance feasibility, resources, and adequate dosimetric information.

In recent years, much effort has been made to improve the accuracy of image analysis and quantification [5]. However, the step from a set of activity data to absorbed dose passes through time points integration: if data are few and do not properly span the radionuclide renal clearance time, most of the time-activity curve is obtained by extrapolation, and, as a consequence, this can dramatically affect the results.

 The issue of experimental-point fitting has been addressed by Glatting and coll in blood serum dosimetry [2], where collecting several data (generally three) for each kinetic phase is feasible from the cost point of view. The aforementioned reasons (expense of data gathering, slow renal clearance) make this more critical for the renal curves in peptide therapy. This question has also been raised by Konijnenberg [8] and Sandström et al. [5]. A dedicated analysis was presented only in a different scenario, that is, when having whole-body scans at 1 h, 1, 2, 7 d for ^177^Lu therapy only, demonstrating a high impact of the 7th day point [9].

The aim of the present study was to compare the dosimetric results obtained in 58 patients undergoing ^177^Lu therapy when considering (a) SPECT scans at 2(±1), 6(±3), 20(±3), 44(±3), 67(±2) h, (b) when neglecting the 6 h point, (c) and when neglecting the 67 h point. The possibility of a simplified Injected Activity clearance IA(*t*) = IA_0PT_ · exp⁡^−〈*λ*〉·*t*^, 〈*λ*〉 obtained fitting all sets of data together and the patient-specific IA_0PT_ (IA at *t* = 0) was also investigated. The impact of time-activity interpolation method (trapezoids, sum of exponentials) on dose and BED estimate was also highlighted, as well as the possible influence of risk factors and of the peptide (DOTATOC versus DOTATATE). The analysis was also extended to ^90^Y, *mutatis mutandis*. 

## 2. Materials and Methods

### 2.1. Patients and Radiopharmaceutical Administration

The cohort included 28 patients (age 46–82, mean 65 yrs.) undergoing ^177^Lu/^90^Y-DOTATATE therapy and 30 patients (age 31–77, mean 58 yrs.) ^177^Lu/^90^Y DOTATOC therapy. During the first cycle patients were evaluated for dosimetry with ^177^Lu DOTATATE (median [range]: 5.0 [3.5–5.7] GBq) and ^177^Lu DOTATOC (5.7 [3.7–7.8] GBq), respectively. All patients received the therapeutic administration of radiolabeled peptides with an infusion of aminoacid solution for renal protection. Details on the synthesis and the administration procedures are described elsewhere [10, 11].

Patients had been diagnosed with metastatic neuroendocrine tumors (primary site: 11 pancreas, 14 gastrointestinal tract, 7 lung, and 3 of unknown origin). The remaining were mainly affected by iodine negative thyroid carcinomas. 

The therapy schedule alternated cycles of ^177^Lu (5.3 GBq/cycle on average) and ^90^Y (2.50 GBq/cycle on average) radiolabelled peptides, for a total of 5 cycles at maximum, 8–10 weeks apart. The physician determined the specific activity to be administered, according to the dosimetric results, patient's clinical conditions, and presence of risk factors for the kidneys (blood hypertension, diabetes) [12]. Toxicity was recorded throughout all the study and up to 6 months after completion. A comprehensive description of this clinical study and patients is reported in a companion paper [13].

### 2.2. Imaging

#### 2.2.1. SPECT-CT Patient Acquisitions

In order to determine the absorbed dose to the kidneys, all patients performed a series of SPECT-CT scans of the abdomen (Symbia T2, Siemens, Germany), at Arcispedale S. Maria Nuova, Reggio Emilia, taken at 2(±1), 6(±3), 20(±3), 44(±3), 67(±2) h after injection. For the sake of simplicity we will refer to these times as 2 h, 6 h, 1 d, 2 d, 3 d in the following.

Acquisitions were performed with a 128 × 128 matrix, zoom = 1,32 × 2 views, 30 s time/view, medium-energy general-purpose collimators. The energy windows were centered over ^177^Lu photon peaks (208 keV and 113 keV, width 15%) while scatter fraction was evaluated with the triple energy window method, through three scatter windows next to the peaks, defined as lower scatter windows (width 10% and 15%) and upper scatter window (width 8%). The SPECT projections were reconstructed by an iterative algorithm with compensations for attenuation, scatter, and full collimator-detector response (Flash 3D iterative algorithm: 10 iterations; 8 subsets; 4.8 mm cubic voxel).

#### 2.2.2. SPECT-CT Calibration and Activity Quantification

To convert counts/s into activity in volumes of interest, the scanner was calibrated by means of a hollow anthropomorphic Torso phantom (Data Spectrum Corporation, Hillsborough, USA) with a set of hollow spheres (volume: 1.5, 0.6, 0.3 mL) and other home-made inserts (two Eppendorf microtubes of 1.5 mL, two conical tubes of 50 mL). Phantom background, liver, and inserts were filled with different activity concentrations of ^177^Lu (conical tubes: 0.8 and 1.2 MBq/mL; spheres with volume of 1.5, 0.6, 0.3 mL: 0.6, 0.75, and 2.5 MBq/mL, resp.; both microtubes: 7 MBq/mL, liver = 0.047 MBq/mL, and phantom background = 0.011 MBq/mL). One microtube and the 0.6 mL sphere were fixed inside the liver region. The acquisition setting and reconstruction algorithm were as described previously (for patients).

The objects were contoured on the CT image, and for each region of interest (ROI), the total counts were divided by the activity, the number of voxels, and the duration of SPECT-CT acquisition to obtain the calibration factor. 

The experimental data representing counts/s/voxel/MBq versus volume (cm^3^) were fitted by the equation *y* = *a*_1_ − *a*_2_ · exp⁡(−*k* · *x*). This curve was used to account also for the partial volume effect when converting counts/s into activity in volumes of interest differently sized. Regards patient kidney volumes, the partial volume effect correction was considered negligible, as from our recovery curves (not shown). Kidney volumes were manually measured on CT scan, and the counts inside each ROI averaged over the number of renal voxels.

 The activity quantified in kidneys was corrected for physical decay to have biological time-activity curves expressed by means of %IA(*t*), the fraction of the total injected activity versus time.

#### 2.2.3. Calculation of Kidney Time-Integrated Activity and Dose

For each patient, time-integrated activity per unit activity ($\tilde{a}$-expressed in h) for the kidneys was computed from experimental %IA(*t*) by means of seven different methods:

1. trapezoidal method up to the last experimental data plus physical decay after the 3 d point (hereafter called TR_ph_);
2. trapezoidal method up to the last experimental data plus monoexponential decay after the 3 d point obtained passing through the last two points (TR_exp⁡_);
3. biexponential fit of the experimental data (BI): *y*(*t*) = *a*_1_ · exp⁡(−*λ*_1,BI_ · *t*) + *a*_2_ · exp⁡(−*λ*_2,BI_ · *t*);
4. monoexponential fit of the experimental data (MN): *y*(*t*) = *a* · exp⁡(−*λ*_MN_ · *t*);
5. monoexponential model deriving a unique fit for all the data sets for each radiopeptide (MNfix): *y*(*t*) = *a*_pt_ · exp⁡(−*λ*_MNfix, TOC_ · *t*); *y*(*t*) = *a*_pt_ · exp⁡(−*λ*_MNfix, TATE_ · *t*). This was done in order to evaluate the feasibility of a simplified method for dosimetry in PRRT, based on the best shared fit parameter (*λ*_MNfix_) specific for the peptide (i.e, DOTATOC and DOTATATE) and the patient-specific initial uptake *a*_pt_;
6. the best fitting function among the analytical functions (3), (4), and (5) according to *F*-test (hereafter called FT) (see next section, point (b));
7. the best fitting function among the analytical functions (3), (4), and (5) according to the visual choice of trained physician/physicists (VIS) (see next section, point (c)).

All fits for (3), (4), (5) were performed using Matlab 7.7.0, Statistics Toolbox. 

Once $\tilde{a}$ was computed, the absorbed dose per unit of injected activity *A*_*o*_(*D*/*A*_*o*_) of ^177^Lu was obtained as $D/A_{o}=S\cdot \tilde{a}$, where *S* is the self-absorbed dose per nuclear transformation in the kidneys for ^177^Lu.

Similar biodistribution and kinetics for peptides labeled with ^177^Lu and ^90^Y are generally assumed [14], therefore the results obtained with ^177^Lu were extrapolated to ^90^Y, simply substituting physical decay constant *λ* and *S* factor in the computation of $\tilde{a}$ and *D*/*A*_*o*_.

The standard *S* values for the kidney of the OLINDA/EXM software (^177^Lu: 0.29 Gy/GBq/h; ^90^Y: 1.76 Gy/GBq/h) were rescaled for the actual patient kidney mass [15]. 

### 2.3. Comparison of Methods and Statistical Analysis

Different criteria were considered to identify the method providing the most accurate estimate of kidney $\tilde{a}$. 

The two trapezoidal methods (1) and (2) cannot be analyzed from a statistical point of view. Their results were simply compared with the best fit identified by a statistical test and the visual criterion.

(a) Considering the three methods using analytical functions (3), (4), and (5), the coefficient of determination [16] *R* ^2^ given by *R* ^2^ = 1 − SSE/TSS was computed for each fit, where SSE is the sum of the squared residuals SEE = ∑_*i*_(*y*_*i*_ − *y*_*i*, fit_)^2^; TSS = ∑(*y*_*i*_ − 〈*y*〉)^2^; *y*_*i*_ is the experimental IA% at time *i*; *y*_*i*, fit_ is the fitting-extrapolated IA% at each time *i*; and 〈*y*〉 is the mean value of the experimental data.

Although most frequently used within pharmacological and dosimetry papers, indeed this criterion just says how a model fits the data better than a constant function equal to the mean value 〈*y*〉 (see Discussion). For this reason *R* ^2^ is not be a very helpful indicator in assessing nonlinear fit quality, as in our case [16].

(b) To properly identify the best method, the one-tailed *F*-test was used, which is indicated for nested functions [2]. This is the case of BI, MN, and MNfix, MN being a particular case of BI and MNfix, a particular case of MN.

Given a pair of fitting functions, one with “reduced” and the other with “full” parameters, with the corresponding sum of squared residuals SSE_reduced_ and SSE_full_ and the number of Degrees of Freedom as DF_reduced_, DF_full_, the *F* value was obtained by the equation:

(1) F(DFreduced−DFfull,DFfull)  =(SSEreduced−SSEfull)/SSEfull(DFreduced−DFfull)/DFfull.

The *P* value of the *F*-test selects which of the two models is better, the null hypothesis being that the simpler (i.e., the one with lower parameters) model is better. The level of significance was set as *α* = 0.10, so if *P* < 0.1 the simpler model was rejected. Although *α* = 0.05 is more often quoted in the literature, its value is more or less arbitrary [2]; our choice was required to increase the statistical power in this case of quite small sample size (5 points). 

(c) Besides, the statistical criteria, the best function among BI, MN, MNfix describing the experimental data was also chosen visually. This was done by three expert physicians/physicists to give more weight to the clinical evidence about the peptide kinetics in the kidneys especially long after the experimental points.

In addition, all the interpolating functions (except MNfix) were computed skipping either the 6 h or the 3 d point to discern their relevance on the determination of $\tilde{a}$ values. Both the $\tilde{a}$ values, determined by the *F* test (${\tilde{a}}_{\text{FT}}$) and visually (${\tilde{a}}_{\text{VIS}}$), were only evaluated in the five-point case.

Finally, the equal-variance one-tailed *t*-test was applied to the two peptide data sets, in order to assess whether the mean time-integrated activity for DOTATOC and DOTATATE was significantly different, despite the interpatient variability, as previously derived in an intrapatient study of 7 patients [4]. To this, a *P* value <0.05 was considered significant, and a *P* value <0.1 rather statistically significant. Similarly, the possible influence of risk factors on $\tilde{a}$ was investigated. 

### 2.4. BED

For radionuclide therapy with an absorbed dose per cycle *D* given in *N* cycles, assuming complete decay and full repair of sublethal damage between cycles, the BED takes the following form [17]:

(2) $$\begin{array}{c} \text{BED}=\text{RE}\cdot D_{\text{TOT}}=\left(1+\frac{G\left(\infty \right)\cdot D}{\alpha /\beta }\right)\cdot ND, \end{array}$$

where *D*_TOT_ = *ND* and RE is the Relative Effectiveness factor, expressed using the Lea-Catcheside factor in the last term, which reduces to

**Figure 1:**
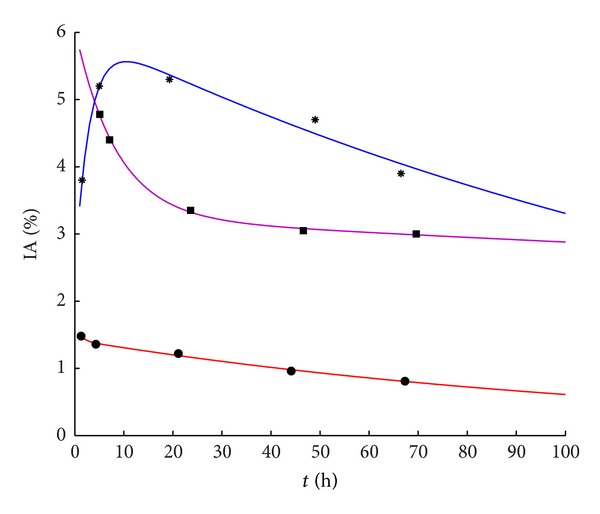
Examples of observed pharmacokinetic behaviour: with-accumulation (blue line, pt no. 45), single-slope clearance (red line, pt no. 43), two-slope clearance (violet line, pt no. 38).

(3) G(∞)  =(a12/λ1(μ+λ1)+2a1a2/(λ1+λ2)(μ+λ1)+2a1a2/(λ1+λ2)(μ+λ2)+a22/λ2(μ+λ2))   ×((a1/λ1+a2/λ2)2)−1,

for a biexponential clearance IA(*t*)/*A*_*o*_ = *a*_1_ · *e* ^−*λ*_1_*t*^ + *a*_2_ · *e* ^−*λ*_2_*t*^ (either *a*_1_ or *a*_2_ could be <0, if an accumulation phase is present (Figure 1)) and to

(4) $$\begin{array}{c} G\left(\infty \right)=\frac{\lambda }{\lambda +\mu_{\text{rep}}}, \end{array}$$

for a monoexponential clearance *A*(*t*)/*A*_*o*_ = *a*_1_ · *e* ^−*λt*^, *μ*_rep_ being the normal tissue repair constant. Following Wessels and coll [18] we set *μ*_rep_ = 0.24 h^−1^ and *α*/*β* = 2.5 Gy for the kidneys. The amount of administered radiopharmaceutical activity varied among patients according to their clinical situation, nonetheless in the following we considered, irrespective of the patient, 4 cycles, 7.4 GBq each for ^177^Lu peptides and 2 cycles, 3.7 GBq each for ^90^Y peptides. This choice allowed interpatient comparison and also comparison with the typical schedules used in the clinic.

BED was only computed for the analytical time-activity fits (methods MNfix, MN, BI), even though a recent work [19] illustrated a procedure for BED computation for piecewise defined fits as well.

For a same patient, relative absorbed dose differences found using diverse fitting functions could result in an amplified difference in BED, which is the dosimetric parameter used, together with absorbed dose, for clinical implementation of treatment planning in PRRT.

## 3. Results

### 3.1. SPECT-CT Calibration

Exponential fit *y* = *a*_1_ − *a*_2_ · exp⁡(−*k* · *x*) of scanner counts as a function of the source volume *x* gave the following sensitivity parameters:*  a*_1_ = 11.4 ± 0.7 counts/s/voxel/MBq, *a*_2_ = 16.5 ± 5.1 counts/s/voxel/MBq, *k* = 0.2 ± 0.1 cm^−3^.

### 3.2. Time-Activity Trends

In the majority of cases (41 out of 58 pts, 71%), the experimental data had a maximum value in the first time point and subsequent values depictable with exponential decrease. A single-slope clearance was observed in 29 pts, while in 12 patients a faster elimination phase followed by a slower one after about 24 h. Conversely, 17 patients showed an accumulation trend until 24 hours after injection. Accumulation was associated with DOTATATE in ten patients and with DOTATOC in seven. 

Figure 1 offers three biological curves which are representative of the three observed pharmacokinetic behaviours.

In all but six cases (90% of pts), the elimination persisted even after 2 d, although at a slower rate as compared to the first day after injection. In particular, the mean (±SD) value of the kidney activity fraction at 3 d showed, on average, a further 20–25% decrease as compared to the 2 d renal uptake (namely for DOTATATE %IA_3d_ = (0.80 ± 0.15) · %IA_2d_, and %IA_3d_ = (0.60 ± 0.20) · %IA_6h_; for DOTATOC %IA_3d_ = (0.75 ± 0.10) · %IA_2d_, and %IA_3d_ = (0.50 ± 0.20) · %IA_6h_).

Table 1 reports the results concerning the biological pharmacokinetic parameters *λ*, obtained by using methods MN, BI, and MNfix. Results are provided separately for DOTATOC and DOTATATE and, with the exception of MNfix, with distinction between cases with accumulation and with clearance only. In 12 cases (21%) biexponential fittings gave *λ*_1_ = *λ*_2_, that is, a monoexponential function in the inspected time interval. Six times a negative *R* ^2^ was found when fitting with accumulation cases with MNfix, meaning that a constant function equal to the mean data value would better fit the data.

Concerning MNfix, the biological half time (*t*_1/2_) was 81 h for DOTATATE and 63 h for DOTATOC; these results are in the middle of the mean MN results: for DOTATATE *t*_1/2_ = 99 h and 69 h patients with and without accumulation, respectively, for DOTATOC *t*_1/2_ = 173 h and 53 h. Concerning the other methods (TRph, TRexp, BI), comparisons are more easily made referring to time-integrated activity $\tilde{a}$.

### 3.3. $\tilde{a}$ Values

**Figure 2:**
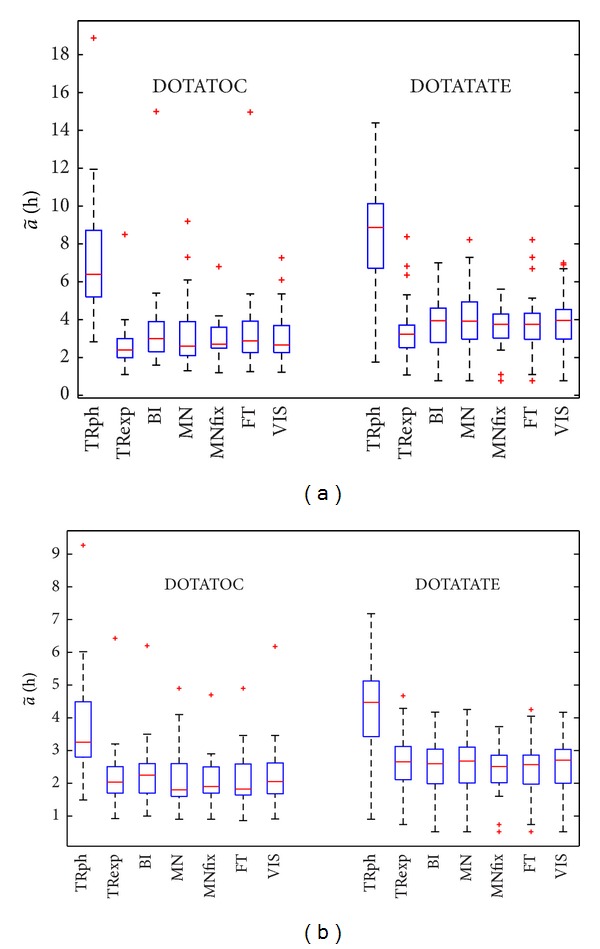
^177^Lu (a) and ^90^Y (b) time-integrated activities $\tilde{a}$ in hours for methods TRph, TRexp, BI, MN, MNfix, VIS, FT. Boxes draw the 25th percentile (lower box bound, indicating 25th of data fall below it), 50th percentile (i.e., median value), and 75th percentile (upper box bound). Crosses indicate outliers defined as observations out of 1.5 · (75th percentile value −25th percentile value). Whiskers extend to the most extreme values that are not outliers.

Box and whisker plots in Figure 2 illustrate the $\tilde{a}$ values for all the methods applied to ^177^Lu peptides (Figure 2(a)) and ^90^Y peptides (Figure 2(b)), with distinction made between DOTATOC and DOTATATE.

The graph emphasizes that ^177^Lu time-integrated activities are consistently overestimated by TRph and slightly underestimated by TRexp as compared to BI, MN, and MNfix. Smaller differences concern ^90^Y because of the minor influence from the curve tail in consequence of the smaller physical half-time *T*_1/2_ (64.1 h for ^90^Y versus 164.2 h for ^177^Lu). The mean ratio between the tail contribution to $\tilde{a}$ $\left({\tilde{a}}_{\text{tail}}\right)$ (i.e., the area under the time-activity curve from last experimental data point on) and the whole $\tilde{a}$ is reported in Table 2, for all the different methods. These ratios point out that the curve after the last experimental point takes about 40% of the total $\tilde{a}$ for ^177^Lu peptides and 25–30% for ^90^Y peptides, while for TRph the ${\tilde{a}}_{\text{tail}}$ accounts for ∼70% (Lu) and ∼55% (Y) of the total $\tilde{a}$.

**Figure 3:**
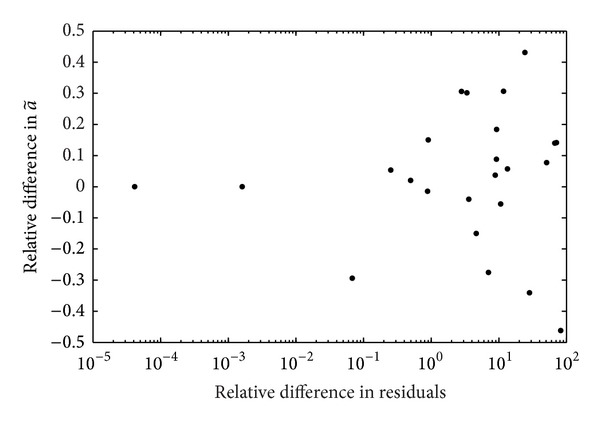
Relative difference of $\tilde{a}$ values for ^177^Lu-DOTATATE between MN and BI methods $y=({\tilde{a}}_{\text{MN}}-{\tilde{a}}_{\text{BI}}\;)/{\tilde{a}}_{\text{BI}}$ plotted against the relative difference of the squared residuals SSE *x* = (SSE_MN_ − SSE_BI_)/SSE_BI_.

Figure 3 points out whether it is possible to univocally identify a “best” $\tilde{a}$ through the analysis of the residuals. MN and BI methods are compared, with the relative difference of the $\tilde{a}$ values $\left(\Delta \tilde{a}=[{\tilde{a}}_{\text{MN}}-{\tilde{a}}_{\text{BI}}]/{\tilde{a}}_{\text{BI}}\right)$ plotted against the relative difference of SSE (Δ*S* = [SSE_MN_ − SSE_BI_]/SSE_BI_). Points are spread out, showing no correlation between $\Delta \tilde{a}$ and ΔSSE. Thus, two curves with similar residuals could lead to very different $\tilde{a}$ values, in other words the best $\tilde{a}$ could not be identified.

### 3.4. Best $\tilde{a}$

The $\tilde{a}$ values of the best method indicated by the *F*-test (${\tilde{a}}_{\text{FT}}$) and those identified by visual analysis (${\tilde{a}}_{\text{VIS}}$) were considered as reference.

**Figure 4:**
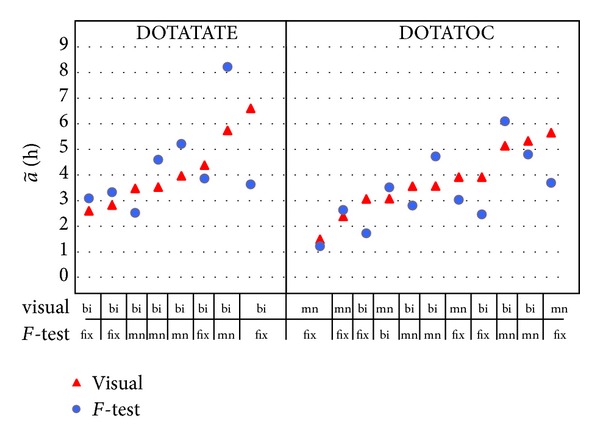
^177^Lu ${\tilde{a}}_{\text{FT}}$ and ${\tilde{a}}_{\text{VIS}}$ for the cases in which there was a discrepancy greater than 10% between the visual and the *F*-test (8 cases for TATE, 11 for TOC).

The *F*-test preferred monoexponential methods in most cases (MNfix: 50%, MN: 38%), while BI in only 12% of cases. According to the visual analysis, the MN was chosen in 50% of cases, and BI was selected in the 50% left (see Figure 4 for the ^177^Lu cases in which the discrepancy among FT and VIS was larger than 10%). Although close to the MN in 21% of cases, the MNfix was avoided in general and definitely considered inappropriate in 79% cases (46 over 58).

For ^177^Lu, in 24% cases (14 over 58), MN discrepancies of $\tilde{a}$ with the best visual model were larger than 20% (reaching even 70%), while for ^90^Y discrepancies higher than 20% were seldom found (3 out of 58 cases only), the largest discrepancy being 25%.

### 3.5. $\tilde{a}$ Comparison

Table 3 reports the mean ± SD values of the ratio—case by case—between the $\tilde{a}$ values from methods TRexp, BI, MN, and MNfix and the ${\tilde{a}}_{\text{FT}}$ and ${\tilde{a}}_{\text{VIS}}$ values. Mean ratios are close to 1, in agreement with the reference method (*F*-test or visual), although SD values are not negligible especially for ^177^Lu peptides, reaching 0.20–0.30 (^177^Lu) and 0.10–0.15 (^90^Y).

Table 3 reports also the results obtained excluding either the* experimental point* at 6 h or at 3 d.

Regarding the TRexp method, the 6 h point has negligible impact, while the lack of the 3 d point causes a remarkable underestimation versus both the *F*-test and the visual results: for example, for ^177^Lu peptides, the mean ratio ${\tilde{a}}_{\text{TRexp}}/{\tilde{a}}_{\text{VIS}}$ of ∼0.7 indicated a mean 30% underestimate $\left({\tilde{a}}_{\text{TRexp}}/{\tilde{a}}_{\text{VIS}}=0.72\pm 0.16\right)$.

At first glance, MN and MNfix may seem to perform similarly on average, the $\tilde{a}$ ratios with the FT and VIS methods being quite close (SD ~0.20 for Lu, ~0.10 for Y). Actually, we found that in 9 cases MNfix gave a result closer to $\tilde{a}$ VIS than the one obtained by MN. However, this might just be due to chance, as the quality of the MN fit, having one parameter more, must be better than the MNfix one (this emerges looking at *R* ^2^ and SSE values in Table 1). This unintended occurrence biased the MNfix results, whose SD would have been worse than MN otherwise.

Excluding TRexp, concerning ^177^Lu, it should be noted that standard deviations are around 0.20 in all situations, regardless of the analytical function considered and of the number of data points –5 versus 4 (see Table 3). This suggests that the gathered data are either inadequate or not properly temporally placed. Conversely, concerning ^90^Y the method used and the 6 h datum have a very negligible influence on $\tilde{a}$; in addition, if the 3d datum is lacking, SDs are higher (around 0.15). 

**Figure 5:**
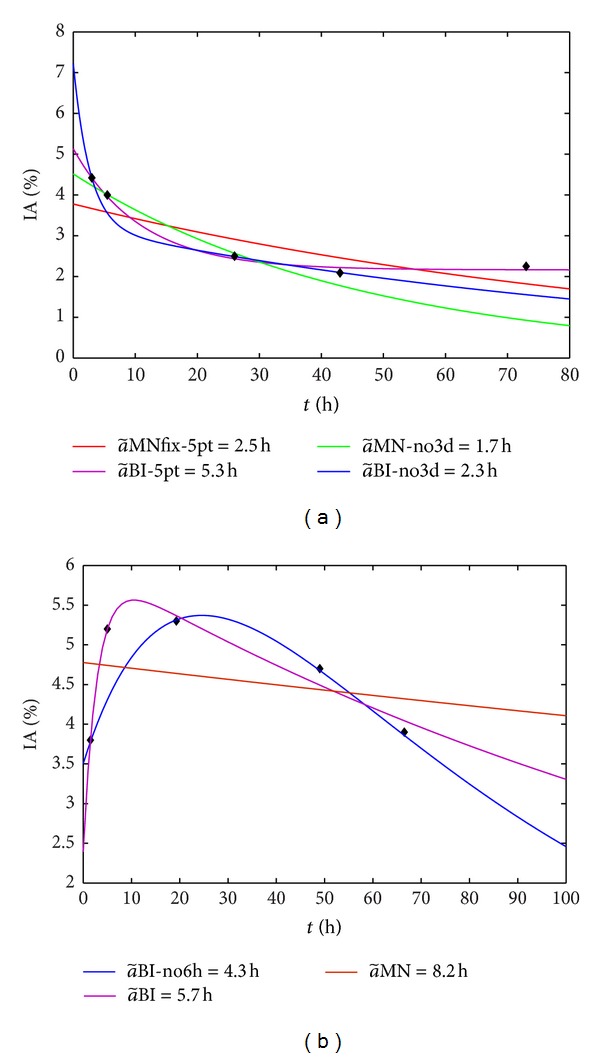
(a) pt no. 35 ^177^Lu DOTATATE: MNfix and BI obtained with all experimental points (red and violet lines, resp.) and MN and BI skipping the 3 d point (green and blue lines, resp.); (b) pt no. 45 ^177^Lu DOTATOC: MN and BI obtained with all experimental points (red and violet lines, resp.) and BI skipping the 6 h point (blue line).

The influence of the 6 h and 3 d points on the fits is shown in Figure 5 by means of the datasets of two patients. For these specific examples, biexponential fits are the best according to the visual method, while *F*-test would have preferred the MNfix method for (a), the MN for (b). For the same patient, ${\tilde{a}}_{\text{BI}}$ varies according to the following ratios: ${\tilde{a}}_{\left(\text{no}\;6\text{h}\right)}/{\tilde{a}}_{\left(5\text{points}\right)}=0.75$; ${\tilde{a}}_{\left(\text{no}\;3\text{d}\right)}/{\tilde{a}}_{\left(5\text{points}\right)}=0.44$.

### 3.6. Impact of Different Kinetic on BED

**Figure 6:**
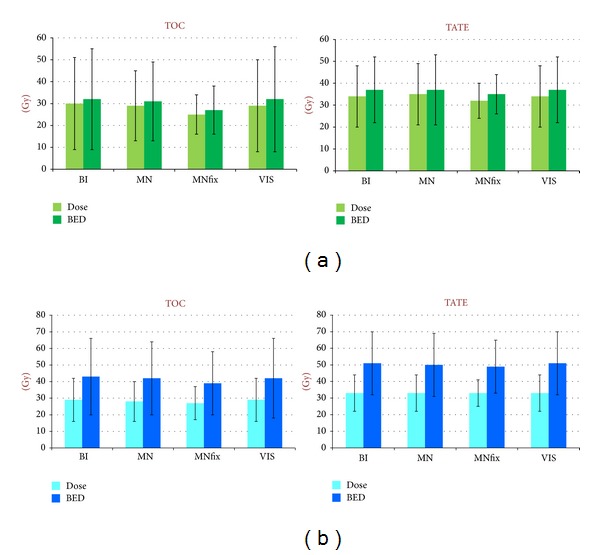
*D* and BED in case of ^177^Lu therapy (upper panels) and ^90^Y therapy (lower panels). The histograms report mean and 1 SD. ^177^Lu and ^90^Y-therapy administer a similar total dose for the specific schemes considered (29.8 GBq in 4 cycles versus 7.4 GBq in 2 cycles, resp.). Conversely, BED is noticeably greater for ^90^Y because of the lower fractionation. Mean ± SD values are similar, but relevant differences among patients can be found (see also Figure 4).

Relative differences on $\tilde{a}$ due to different methods extend to *D* and BED.  Figure 6 reports *D* and BED to the kidneys calculated with methods BI, MN, MNfix, VIS, for ^177^Lu and ^90^Y, respectively. *F*-test results are neglected because they are similar to VIS results on average (see Figure 2).

Regards the absorbed dose, intermethod variations are the same as those found for $\tilde{a}$ (see $\tilde{a}$ *comparison*). Concerning the BED, it is remarkable that for ^177^Lu the relative effectiveness factor (RE) for each patient is almost the same irrespective of the method (BI, MN, or MNfix) by means of computation. For ^177^Lu the relative variation of RE for both MN and MNfix compared to BI (mean ± SD) is 1 ± 1% with a maximum of 5%, for ^90^Y is 4 ± 4% with a maximum of 22%. 

For ^177^Lu, the mean RE is barely 1.1 with a narrow inter-patient variability range (∼0.05), while for ^90^Y it is about 1.5 with a greater variability (∼0.30). 

### 3.7. Influence of the Peptide and of Risk Factors

Irrespective of the radionuclide and of the method used for computation, the time-integrated activity in kidneys, as well as *D*, was significantly higher for DOTATATE (*t*-test: *P* = 0.008). This is in agreement with Esser and coll [4] whose finding showed for the same patient undergoing ^177^Lu therapy, $\tilde{a}$ (DOTATATE) = $1.4\cdot \tilde{a}$ (DOTATOC). It should be noted that regarding tumor $\tilde{a}$, they found $\tilde{a}$ (DOTATATE) = $2.1\cdot \tilde{a}$ (DOTATOC). However, we were not concerned with tumor dosimetry in this study.

According to the visual choice, for ^177^Lu, the mean ± SD value of $\tilde{a}$ was 3.9 ± 1.4 h for DOTATATE and 3.2 ± 1.2 h for DOTATOC. The corresponding absorbed dose values were 1.0 ± 0.2 Gy/GBq and 0.7 ± 0.2 Gy/GBq.

For ^90^Y, $\tilde{a}$ was 2.7 ± 0.9 h for DOTATATE and 2.1 ± 0.7 h for DOTATOC and absorbed doses 3.7 ± 1.5 Gy/GBq and 2.9 ± 1.3 Gy/GBq, respectively.

**Figure 7:**
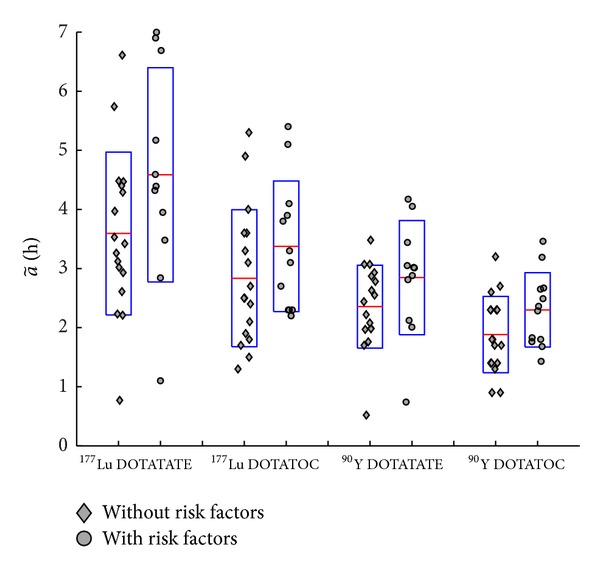
Time-integrated activity per unit activity $\tilde{a}$ for patients with risk factors (RF) (diamonds) and without (NRF) (dots). For each population, the mean value is indicated by the horizontal line, and the box extends to mean ± SD. For DOTATATE, 11 patients were RF and 17 NRF, and the *P*-values of *t*-test were 0.05 and 0.06, for ^177^Lu and ^90^Y, respectively. For DOTATOC, 12 patients were RF and 17 NRF (one outlier was excluded), and *P*-values were 0.09 and 0.06, for ^177^Lu and ^90^Y, respectively.

The *t*-test analysis highlighted a rather statistically significant difference between the mean $\tilde{a}$ values for patients with (RF) and without risk factors (NRF) (one-tailed *t*-test: *P* < 0.10). The RF cohort on average received a *D* 1.20–1.25 higher than the NRF one (Figure 7). An increased number of patients could confirm this explorative finding with a higher level of significance (e.g., *P* = 0.05).

## 4. Discussion

Tailoring peptide receptor radionuclide therapy (PRRT) according to dosimetry has been shown to be of great value in clinical practice and should replace the criteria of administering a fixed of radioactivity amount or an activity correct for the patient body weight or surface [20]. 

Besides the red marrow, it is the kidneys that are the critical organs in this therapy, especially with ^90^Y-peptides [1, 3, 21]. Much effort is being put into improving dosimetric accuracy in the quantification of activity: correcting *S* factors for patient-specific kidney mass [22], using SPECT instead of whole-body images [5], taking into account scatter and attenuation correction [23], including collimator response correction [7], computing a 3D activity distribution [6] to consider the equivalent uniform dose (EUD) instead of the mean absorbed dose. 

Conversely, the impact of kinetics and image timing on the absorbed dose estimate specific for the kidneys has been neglected to a certain extent.

It has been stated [5] that a margin of error of 20% or less would be of great value when evaluating normal tissue complication probability (NTCP) [24]. 

In this work we have shown that data-collection/image-acquisition timing and the method for data interpolation cannot be overlooked, since calculation of absorbed dose could lead to errors even greater than 20% and greater than those originating from activity quantification. This is not acceptable, especially because such errors can be easily avoided by an adequate kinetic analysis. 

Concerning the two issues of data-acquisition timings and interpolation, several dosimetric approaches exist in clinical practice, as shown in Table 4.

According to the EANM guidelines on internal dosimetry [26] three measurements for each kinetic phase are needed. Because the kinetics of DOTATOC and DOTATATE peptides in the kidneys are often associated with more than one phase—generally two—this would be demanding for patients as well as clinicians, since the acquisition of at least six images is difficult and time consuming. Consequently, finding a compromise that optimally balances accuracy and feasibility is of the utmost importance. The current situation for most clinical studies, in which no more than five points are available, is reflected in Table 4.

The choice of many investigators [5, 7, 9] has been to focus only on the slower phase which starts after 24 h, because this takes more than 70% of total time-integrated activity. Sandstrom pointed out that doing so there could be an overestimation, when extrapolating the first 24 h part. According to our results, a slight underestimation could be possible as well. 

In our cohort we stopped data collection 3 days after therapy for logistic reasons, as it is cumbersome for most patients to stay one whole week in the proximity of the hospital. 

Our data showed that the method used to compute $\tilde{a}$ is crucial if data collection is stopped when the remaining administered activity is higher than 30% of the activity present in the kidneys at time zero; in contrast, we found that a careful choice of the method (excluding trapezoidal methods) results in negligible importance (differences smaller than 10%) when data reach two radionuclide effective half-lives. Moreover, in this situation the 6h point could be spared. This finding therefore illustrates that the use of radionuclides with half-life shorter than *t*_1/2eff_ (^177^Lu/^90^Y), like the *β* ^+^-emitter ^86^Y [3], is not advisable for PRRT dosimetry. For example, taking a median biological decay constant of *λ* = 0.01 h^−1^ (Table 1), *t*_1/2eff_ (^90^Y) ∼35 h, *t*_1/2eff_ (^177^Lu) ∼50 h, it would turn out that data are needed up to ∼70 h for ^90^Y peptides and ∼100 h for ^177^Lu peptides. If this is not possible, it must be pointed out that the obtained $\tilde{a}$ values could differ up to 70% depending on the method used for their calculation and the acquisition timings. In particular, a unique interpolating function cannot be used without taking into consideration the specific individual kinetics, because the weight of the tail becomes of major relevance. Thus, the question of choosing the best method among the several available (exponential functions, trapezoidal methods) arises. Moreover, establishing a principle for the selection of the most appropriate model allows an increased reproducibility of the results, as the user dependence is reduced [2]. 

Several criteria exist to choose the preferable model, but the small number of data points (five) is a major drawback in our study. The modified Akaike information criterion (AIC) proved to be an efficient approach [2] in blood serum dosimetry for radioimmunotherapy with anti-CD66 antibody, but in our case, it cannot be applied because of the small sample size (*N*) as compared to the number of parameters (*K*), four in a biexponential function, as the condition for its use (*N* > *K* + 2) does not apply. In our scenario, the AIC could be only used to evaluate the model most supported by the data for the whole data set, not for each single patient [27]. 

The *F*-test is feasible in principle, bearing in mind that the statistical power is low though (i.e., the probability density function *F* is broad). Moreover, these tests (*F*-test, AIC) are based on the residuals between the fitting function and the measured data which stop at 3 days p.i., when 30%–40% of the activity reaching the kidneys at time zero is still not decayed (and in this way considered just by extrapolation): the great impact of the tail led to the paradox that on several occasions MNfix performed better than MN, although the former has one degree of freedom less (see $\tilde{a}$ *comparison*). For this reason it is important to use all the available information—theoretical and empirical—in the model selection, besides the statistical criteria [2], which is why we put side by side the best models as determined by the *F*-test and visually, the latter is being used, as it was, as “reference.” 

It is important to remember that the determination coefficient (*R* ^2^), although commonly used, is not actually a very useful indicator when using nonlinear regression functions [16], because it compares the sum of residuals with the distance of the data from their mean (*R* ^2^ = 1 − SSE/TSS; see *comparison of methods and statistical analysis*). When data are distanced from their mean value, as most often occurs in exponential curves, *R* ^2^ could be a misleadingly high parameter. 

Despite all the limitations described previously for the various candidate criteria with so few data points (five, as generally available in practice), useful information could be gained analyzing our data.

- *TRph*: the TRph cannot be applied with physical only decay starting from the 3 d point (or even before), because the physiological clearance in 52 on 58 cases was found after that; moreover, some investigators [5, 9] found biological clearance even up to 7 days (i.e., at 7 d, the measured activity was from 20% to 30% that of the one at 1 h and from 25% to 40% that of the one at 1 d). Pursuing this way, the overestimation of $\tilde{a}$ is evident and is marked (Figure 2). Our interest in this method was drawn after its implementation in commercial software for dosimetry [28].
- *TRexp*: concerning TRexp, it comes to light that fitting a monoexponential function by means of only two points is not a reliable choice: $\tilde{a}$ is very dependent on which ones are being used. For example we found that using the 1-2 d points instead of the 2-3 d for extrapolation, $\tilde{a}$ decreased on average of 18% for either ^177^Lu and ^90^Y. Therefore, this method is not recommended when experimental data span is less than two effective radionuclide half-lives.
- *MNfix*: trying to use a single *λ* for all patients did not give satisfactory results for ^177^Lu, as differences with the best visual model were ±20% (and could even be worse, because it was shown that in 9 cases the ${\tilde{a}}_{\text{MNfix}}$ value was close to ${\tilde{a}}_{\text{VIS}}$ just by chance). Conversely, for ^90^Y it was not to be rejected (the mean ratio between the best ${\tilde{a}}_{\text{VIS}}$ and ${\tilde{a}}_{\text{MNfix}}$ is 0.97 ± 0.11, with a maximum discrepancy of 35%). This is explained because of the lower effective half-life of ^90^Y as compared to ^177^Lu. It must be remembered that in the MNfix method the decay constant *λ* was fixed, but the initial activity was patient specific.
- *MN and BI*: for ^177^Lu a monoexponential function is a safe choice only having taken data after 24 h and reaching two effective radionuclide half-lives; conversely, when data are available from the injection up to 2-3 days a biexponential function is highly recommended (above all if accumulation or fast clearance is observed during the first day), as in this case the ratio of $\tilde{a}$ for the monoexponential over the best visual model is 1.02 ± 0.22, while with the biexponential model is 1.02 ± 0.07; that is, BI has a lower SD meaning a better agreement.
- *VIS*: when radio-peptide therapy cannot rely on the benefit of a dosimetry-based planning, Figure 7 might be of some help, providing the mean ± SD of the best $\tilde{a}$ values calculated from the visual method, for DOTATOC and DOTATATE peptides and ^177^Lu and ^90^Y radionuclides. Regarding 7.4 GBq ^177^Lu DOTATATE administration, our findings are consistent with those from Sandstrom and coll, who reported an absorbed dose to the kidneys of 5 ± 2 Gy in a 24-patient cohort (cfr [5], *small VOI method*, mean value between left and right kidney *D*), to be compared with 6 ± 2 Gy of our 25-patient cohort, having excluded 3 outlier patients with doses of about 14 Gy/cycle.
- *Risk factors*: it was recently shown [29] that impairment in renal function assessed by glomerular filtration rate led to higher mean kidney absorbed doses. We found that risk factors (hypertension, diabetes, lesions close to renal parenchyma, etc.), which are an evidence of renal impairment, led to a statistically significant increase in absorbed dose to the kidneys. In our cohort, patients with risk factors (RF) on average received a dose 1.20–1.25 times higher than patients without. This finding should be considered together with the clinical evidence [12] that the maximum tolerable BED is lower for patients with RF than without RF (28 Gy vs 40 Gy).
- *BED*: finally, to take into account the effect related to the dose rate and to the response of the irradiated tissue, planning treatments are conducted constraining the BED as well [12, 17]. We found that for ^177^Lu using formula (3) even with a fixed *G*(*∞*) value (i.e., obtained from formula (4) with *λ*_fix_) does not give a dissimilar result if the more complicated *G*(*∞*) of formula (3) is being used. To give an example, the mean ratio of the RE values using the *G*(*∞*) for MNfix and BI functions is 1.01 ± 0.01. In other words, what is important in the estimation of the BED for ^177^Lu is the dose calculation accuracy only (besides the reliability of the *α*/*β* coefficient, obviously). For ^90^Y, the same RE ratios were slightly greater, leading to a mean value of 1.04 ± 0.04.

## 5. Conclusions

Accurate dosimetry is mandatory to fully exploit the potential of PRRT. Nonetheless, not only it does make high demands on the level of staff commitment and facilities, but also on the patients themselves who in most cases can be discharged two days after therapy. 

We found that if data are not available up to two effective half-lives (∼4 days for ^177^Lu, ∼3 days for ^90^Y), the estimation of kidney absorbed dose is consistently influenced by the interpolation method: concerning MNfix, MN, and BI, in 30% of cases for ^177^Lu and 20% of cases for ^90^Y differences between methods were higher than 10%, reaching 60% and even higher when considering also TRph and TRexp. 

A monoexponential clearance with an averaged *λ* could be used in exceptional cases in ^90^Y therapy, whereas for ^177^Lu is totally inadequate. Concerning BED, RE is almost insensitive to the analytical time-activity curve being used, differences between analytical methods being within 10%. 

The use of DOTATATE instead of DOTATOC caused a D increase of 1.25–1.30 (*t*-test, *P* < 0.05), while the effect on tumor was not evaluated. The risk-factor group had a D increase of 1.20–1.25 compared to the group without risk factors (*t*-test, *P* < 0.10).

## Figures and Tables

**Table 1:** Statistical results (*R* ^2^ and SSE) and *λ*_*s*_ computed by means of different fitting functions, with distinction between DOTATOC and DOTATATE peptides, with and without accumulation behaviour. Concerning *λ*_MNfix_, a single value for each peptide was computed, irrespective of the kinetic behaviour. A negative *R* ^2^ (obtained in 6 MNfix fits) means that a constant function equal to the mean value of the data would fit better; that is, the fit is of very poor quality.

	Biological constants *λ*					
	Cases with accumulation			Clearance only cases		
	Mean ±1SD · 10^−3^	*R* ^2^ median	SEE median	Mean ±1SD · 10^−3^	*R* ^2^ median	SEE median
	(range) (h^−1^)	(range)	(range)	(range) (h^−1^)	(range)	(range)
*DOTATATE *						
*λ*_MNfix_	8.6	0.71 (−1.6–0.99)	0.89 (0.07–5.30)	8.6	0.81 (0.40–0.99)	0.58 (0.004–4.04)
*λ*_MN_	7 ± 3 (2–13)	0.82 (0.08–0.98)	0.59 (0.007–1.83)	10 ± 4 (5–21)	0.94 (0.57–0.99)	0.25 (0.002–1.07)
*λ*_1BI_	207 ± 147 (42–427)	0.99 (0.75–1)	0.017 (0.001–0.147)	47 ± 9 (9–311)	0.98 (0.75–0.99)	0.092 (0–1.057)
*λ*_2BI_	11 ± 5 (6–21)			16 ± 37 (0–162)		

*DOTATOC *						
*λ*_MNfix_	11.04	−0.71 (−1.47–0.60)	2.32 (1.05–11.24)	11.04	0.82 (0.36–0.98)	0.49 (0.05–9.09)
*λ*_MN_	4 ± 2 (1–7)	0.57 (0.01–0.93)	0.34 (0.13–4.71)	13 ± 5 (7–24)	0.97 (0.66–1.00)	0.17 (0.002–5.26)
*λ*_1BI_	191 ± 114 (28–308)	1 (0.89–1)	0.01 (0–0.02)	143 ± 155 (9–443)	0.99 (0.80–1)	0.025 (0–0.364)
*λ*_2BI_	12 ± 5 (6–22)			10 ± 5 (0–22)		

**Table 2:** The mean fraction of time-integrated activity per unit activity $\tilde{a}$ situated after the last experimental datum $\left({\tilde{a}}_{\text{tail}}/\tilde{a}\right)$ is reported for the methods TRph, TRexp, BI, MN, MNfix, VIS, FT.

${\tilde{a}}_{\text{tail}}/\tilde{a}$	TRph	TRexp	BI	MN	MNfix	FT	VIS
^ 177^Lu TOC	0.73	0.29	0.40	0.38	0.45	0.43	0.38
^ 177^Lu TATE	0.73	0.34	0.42	0.43	0.35	0.38	0.41
^ 90^Y TOC	0.56	0.28	0.26	0.24	0.39	0.33	0.25
^ 90^Y TATE	0.57	0.32	0.27	0.28	0.28	0.28	0.26

**Table 3:** Ratio of $\tilde{a}$ computed with BI, MN, MNfix, TRexp methods with best $\tilde{a}$ determined by means of *F*-test and visual choice. “6 h-point excluded” and “3 d-point excluded” mean that fitting was conducted skipping the 6 h and the 3 d point, respectively. The shared *λ* (MNfix) was only computed in the 5-point case.

Mean ± 1SD	$\tilde{a}$ ratios					
		^ 177^Lu peptides			^ 90^Y peptides	
	5 points	6 h point excluded	3 d point excluded	5 points	6 h point excluded	3 d point excluded
*F-test *						
BI/FT	1.10 ± 0.29	1.09 ± 0.29	1.08 ± 0.21	1.04 ± 0.10	1.03 ± 0.09	1.04 ± 0.09
MN/FT	1.05 ± 0.16	1.04 ± 0.17	1.09 ± 0.31	1.02 ± 0.06	1.03 ± 0.07	1.03 ± 0.13
MNfix/FT	1.00 ± 0.18	Na	Na	0.99 ± 0.10	Na	Na
TRexp/FT	0.90 ± 0.30	0.91 ± 0.31	0.78 ± 0.18	1.07 ± 0.22	1.09 ± 0.22	0.97 ± 0.16

*Visual *						
BI/VIS	1.02 ± 0.07	1.02 ± 0.19	1.03 ± 0.18	1.01 ± 0.05	1.00 ± 0.06	1.01 ± 0.09
MN/VIS	1.02 ± 0.22	1.00 ± 0.23	1.05 ± 0.36	1.00 ± 0.08	1.00 ± 0.09	1.01 ± 0.16
MNfix/VIS	0.97 ± 0.20	Na	Na	0.97 ± 0.11	Na	Na
TRexp/VIS	0.88 ± 0.24	0.89 ± 0.24	0.72 ± 0.16	1.06 ± 0.20	1.07 ± 0.21	0.90 ± 0.14

**Table 4:** Characteristics of different dosimetric approaches concerning acquisition timing and interpolation method.

Investigator	Therapeutic nuclide	Acquisition timings	Interpolation method
Esser et al. [4], Sandström et al. [5], Larsson et al. [9]	^ 177^Lu	1, 4, 7 d	MN

Hindorf et al. [25]	^ 90^Y	Not fixed, up to 19–48 h, 2–4 acquisitions	Not reported for kidneys

Baechler et al. [17]	^ 90^Y, ^177^Lu, ^111^In	0.5, 4 h, 1, 2 d	MN of last three points

Cremonesi et al. [3]	^ 90^Y, ^177^Lu	1, 4 h, 1, 2, 3 d	MN or BI

Garkavij et al. [7]	^ 177^Lu	1 h, 1, 4, 7 d	TRexp
